# Understanding reasons and determinants of medication non-adherence in community-dwelling adults: a cross-sectional study comparing young and older age groups

**DOI:** 10.1186/s12913-023-09904-8

**Published:** 2023-08-24

**Authors:** Lixia Ge, Bee Hoon Heng, Chun Wei Yap

**Affiliations:** Health Services and Outcomes Research, National Healthcare Group, 3 Fusionopolis Link #03-08, Nexus@one-north, Singapore, 138543 Singapore

**Keywords:** Medication adherence, Non-adherence, Medication management, Chronic illness, Cross-sectional, Community care, Age differences, Risk factors

## Abstract

**Background:**

Medication non-adherence has become a striking problem among patients with chronic diseases worldwide. However, literature on prevalence, reasons and factors associated with medication non-adherence in Singapore general population is still lacking. This study aimed to (1) estimate the prevalence of intentional and unintentional medication non-adherence in young (aged 21–64 years) and older adults (aged ≥ 65 years), respectively; (2) identify and compare the main reasons for non-adherence; and (3) examine the association between potential factors and non-adherence in each group.

**Methods:**

This study sampled 1,528 community-dwelling adults on medications (young adults:766, older adults: 762) from a cross-sectional population health survey conducted in the northern and central regions of Singapore in 2018/2019. Self-reported medication non-adherence and its reasons were collected using a modified questionnaire and compared between the two groups. Multiple logistic regressions were conducted to examine the association between potential factors (e.g., social-demographic factors, smoking and drinking status, presence of diabetes, hypertension, or dyslipidaemia, and presence of depressive symptoms) and medication non-adherence in each group.

**Results:**

The prevalence of non-adherence was 38.4% and 22.3% in young and older adults, respectively, with young adults reporting higher unintentional and intentional non-adherence rates than older adults. “Afraid of developing drug dependence” was the most common reason in both groups (young:74.8% vs. old:73.5%). Compared to young adults (3.7%), “Not understanding medication labels” was more prevalent in older adults (8.8%). Presence of depressive symptoms was associated with non-adherence in both young (odds ratio [95% confidence interval]: 3.00 [1.79, 5.05]) and older adults (4.16 [2.31, 7.51]). Being employed (2.92 [1.76, 4.84]) and taking ≥ 2 medications (1.42 [1.04, 1.95]) had positive association while personal income of SGD1,000–4,000 (0.53 [0.36, 0.77]) and current smoking (0.61 [0.39, 0.95]) had inverse association with non-compliance in young adults. Diagnosis of diabetes, hypertension, or dyslipidaemia (2.63 [1.25, 5.53]) was associated with higher odds of non-compliance in older adults.

**Conclusions:**

Young adults had higher prevalence of medication non-adherence than older adults. The main reasons for non-adherence reported by young and older adults were generally comparable. Presence of depressive symptoms was a risk factor of medication non-adherence in both groups.

**Supplementary Information:**

The online version contains supplementary material available at 10.1186/s12913-023-09904-8.

## Background

Long-term medication treatment, the most fundamental frontline strategy for chronic disease management [1], is commonly associated with patients’ poor adherence to prescribed medications [2]. Medication non-adherence has become a striking problem among patients with chronic diseases worldwide, with an estimated average rate of 50% in developed countries and an even higher rate in developing countries [2]. As medication non-adherence significantly compromises the effectiveness of treatment, it has remarkable impacts on individuals, healthcare system, and society, resulting in suboptimal health outcomes, increased healthcare utilisation and cost, and higher mortality [3–7].

Medication non-adherence is a complex and dynamic behavioural process that is strongly influenced by factors at intrapersonal, interpersonal, service and system levels [8–10]. There has been an abundance of literature identifying the potential factors associated with medication non-adherence for either general chronic conditions [11, 12] or specific chronic conditions, e.g., diabetes mellitus and/or hypertension [5, 13, 14], dyslipidaemia [15], cardiovascular disease [16], and kidney disease [17] both locally and internationally. World Health Organization’s systems model for adherence proposed to categorize the factors into five interactional domains: patient-related factors, social and economic factors, condition-related factors, therapy-related factors, and health care team and system-related factors [18].

Patient-related factors include patients’ function, knowledge, perceptions and beliefs, and attitudes. Both quantitative and qualitative studies have found that a patient’s knowledge of the diseases and medications, beliefs and attitudes towards medication taking, and the effectiveness of symptom control influenced medication adherence [12, 15, 17, 19, 20]. Social and economic factors include patients’ demographics, socioeconomic status, literacy, social support networks, living conditions, affordability of medication, as well as some environment and culture related factors. Several social and economic factors including age, gender, ethnicity, educational level, employment status, and financial status were reported to be associated with medication non-adherence [9, 21, 22]. Condition-related factors represent health or specific disease-related issues encountered, e.g., level of disability, presence and severity of symptoms, and depression or psychotic disorders or other comorbidities. While some studies found that duration of condition was associated with poor adherence [15, 23], a local study observed that older adults with poor self-perceived physical and mental health status, or having more or some specific conditions (e.g., diabetes, hypertension, and chronic heart failure) tended to have higher prevalence of non-adherence, although the difference became non-significant after adjusting for other potential covariates [1]. While some studies reported that smokers or alcohol consumers were less adherent to medication for chronic diseases than their counterparts, the difference was either insignificant or inconsistent [11, 24–26]. Therapy-related factors identified to be associated with poor adherence include complex medication regimens [23], receiving incomplete treatment information [12], difficulty of drug consumption [11], and actual or perceived side effects [1, 11, 18]. The findings on the impact of number of prescribed medications and dosage regimen on non-adherence are inconclusive [1, 15, 23]. The association of health care team and system factors with medication non-adherence is relatively least examined. One study observed that poor prescription instructions by health providers was associated with non-adherence [13]. However, the findings on the impact of financial medication assistance on adherence are controversial [27, 28].

The factors identified to be associated with medication non-adherence from different studies are rather heterogeneous [22]. The inconclusive evidence implies that medication non-adherence may be influenced by varied factors in different populations, especially in the context of developed countries [2] as the impact of a person-level factor on medication taking is influenced by factors in other domains such as interpersonal factors, condition and treatment-rated factors, and system-level factors. Hence, to improve medication adherence of the local community-dwelling adult population with chronic conditions, it is necessary to identify context-specific reasons and factors that attribute to medication non-adherence. However, literature on prevalence, reasons and factors associated with medication non-adherence in local general population is still lacking. Prior studies indicated that young and older adults had different adherence rate [11, 12], thus examining factors associated with non-adherence by age group may provide more in-depth understanding. Hence, we conducted this study to (1) estimate the prevalence of intentional and unintentional medication non-adherence in two groups: young adults (aged 21–64 years) and older adults (aged ≥ 65 years), (2) identify and compare the main reasons for medication non-adherence in two groups, and (3) examine the association between potential factors and medication non-adherence in each group.

## Methods

### Study design and setting

Data of this cross-sectional study were derived from the Phase 2 Population Health Index (PHI) survey, a population health survey conducted among representative community-dwelling adults (aged ≥ 21 years) in the central and northern regions of Singapore between November 2018 and August 2019.

### Study participants and recruitment procedure

The sampling procedure in the central and northern regions of Singapore was conducted separately and independently. A sampling frame of residential dwelling units in the central region was constructed by matching postal codes in the National Database on Dwellings in Singapore maintained by the Department of Statistics with the list of postal codes for the central region. Same approach was taken to construct a sampling frame of residential dwelling units in the northern region. A sample of 7,000 and 7,067 residential dwelling units was selected from the central and northern regions respectively, based on proportionate allocation stratified by the specified planning areas in respective region. Within each planning area, a sample of dwelling units was selected proportionately from the designated broad dwelling type groups (Housing and Development Board (HDB) properties, condominiums and other apartments, and landed properties).

Hardcopy invitation letters were sent by post to 5,271 dwelling units in the northern region which were randomly selected from the 7,000 dwelling units based on the proportionate allocation by the three planning areas and to all the 5,623 dwelling units in the central region. This was to notify the residents that there would be trained surveyors visiting them at their doors for the survey. The Kish grid [29], a method using a pre-assigned table of random numbers to select participants, was used to identify one eligible household member from each selected dwelling unit to participate in the study. By using these sampling procedures, we aimed to ensure the participants were representative of the target population in respective region.

**Fig. 1:**
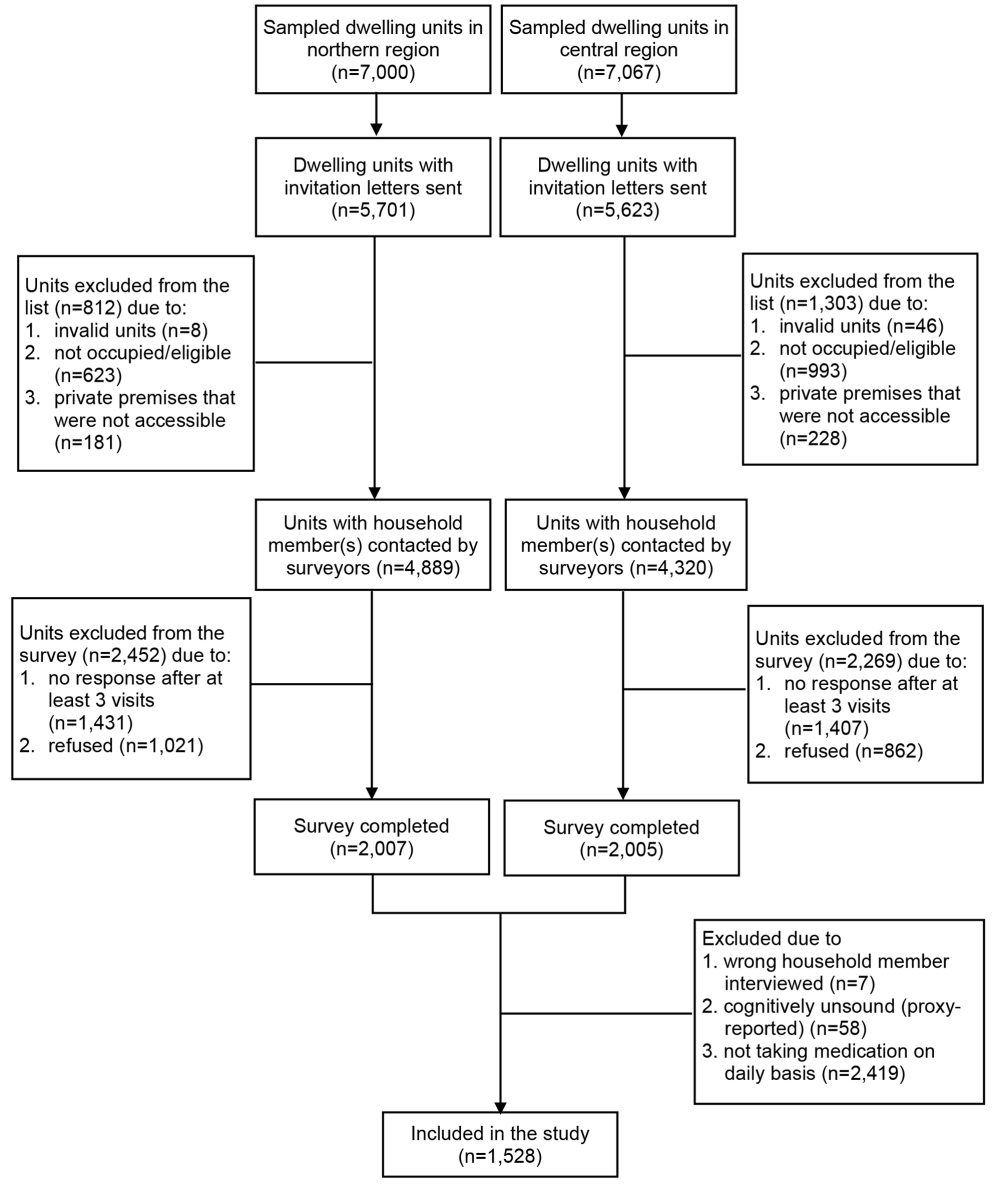
Study participant selection process

Trained surveyors conducted door-to-door visit to check eligibility of the household members and recruit participants. Singapore citizens and permanent residents aged ≥ 21 years and having stayed in the selected household for more than 6 months in past year were eligible for the survey. A dwelling unit was treated as “invalid”, “not occupied/eligible”, and “not accessible” if the block was dismantled, the unit was vacant or rented out to foreigners or the occupiers stayed overseas during the study period, and the unit was a private premise not accessible due to security control, respectively. Hence, they were excluded from the response rate calculation. A dwelling unit was treated as “refused” if either the designated household member or the family member refused participation during the door-to-door visit; and a unit was treated as “not-contactable” if the surveyors were unable to get in touch with any household members after making at least visits at different timing of different days. A total of 2,007 individuals from the northern region and 2,005 from the central region eventually participated in the survey (Fig. 1).

For this study, individuals who were selected wrongly (n = 7), lacking the capacity to give consent or respond to survey questions independently (n = 58), or not taking any prescribed medication (n = 2,419) were excluded from the analysis. Hence, a total of 1,528 participants who were cognitively sound and self-indicated that they were taking at least one prescribed medication on daily basis were included in the analysis.

### Measures

#### Prevalence of medication non-adherence

The prevalence of medication non-adherence was determined by two survey questions: (1) “Some people forget to take their medicines. How often does this happen to you?” and (2) “Some people I have talked to say that they purposely miss out a dose of their medication or adjust it to suit their own needs. How often do you do this?”, representing unintentional and intentional non-adherence, respectively. Each question had four options: “Never”, “Rarely”, “Sometimes”, “Often”, and “Very often”. As people tended to under report the frequency of missed dosing [30, 31], for this analysis, the response of “Never” was coded as “Adherent”, and the other four responses were coded as “Non-adherent”. “Non-adherent” for either question was treated as overall medication non-adherence.

#### Reasons for medication non-adherence

The reasons for non-adherence were collected using a questionnaire with 17 potential reasons (Supplementary Table 1) which were adapted from the Culig Adherence Scale [32] and could be categorized into five domains: patient-related factors, social / economic factors, condition-related factors, therapy-related factors, and health-system related factors [18]. Each reason had four options: “Never”, “Rarely (1–2 times yearly)”, “Sometimes (3–5 times yearly)”, and “Often (> 5 times yearly)”. The frequency of one reason was calculated based on the overall count of the responses of “Rarely”, “Sometimes”, or “Often” to the respective reason.

#### Potential factors examined

The potential factors included for examining relationship with medication non-adherence in each age group were: region (central vs. northern), gender (male vs. female), ethnicity (Chinese, Malay, Indian, or others), personal income level (> SGD4,000, SGD1,001–4,000, or ≤ SGD1,000), living arrangement (living with others vs. living alone), employment status (not employed vs. employed), current smoking status (not smoking vs. smoking based on the responses to the question: “Do you currently smoke tobacco products?”), alcohol abuse which defined as taking 6 or more servings on one occasion (no vs. yes), presence of diabetes, hypertension, and/or dyslipidaemia (DHL; no vs. yes), and number of medications (1 only vs. 2 or more). Presence of depressive symptoms (no vs. yes) was measured by the 9-item Patient Health Questionnaire [33] using the cut-off score of 5 (mild to very severe depression) out of the total score of 27 (each item has a score from 0 to 3).

### Statistical analyses

Descriptive analyses were conducted for the characteristics of participants in each age group with mean and standard deviation (SD) reported for continuous variables, and frequency and percentage (%) reported for categorical variables. Chi-squared tests or Fisher’s exact tests were used to examine the differences in characteristics between the two groups.

The prevalence of overall, unintentional, and intentional medication non-adherence was calculated for the participants in each group and compared using Chi-squared tests. Within each group, the distribution of medication non-adherence across each characteristic group was described. Chi-squared tests or Fisher’s exact tests were conducted to determine the univariate relationship between respective characteristics and medication non-adherence and to examine the difference in distribution for each reason for medication non-adherence in each group. Multiple logistic regression was performed to identify factors associated with medication non-adherence (dependent variable) for individual groups. Due to small number of participants of other ethnic groups, ethnicity was reclassified into two groups: Chinese and non-Chinese. The healthcare of residents living in the central and northern regions of Singapore are generally managed by different healthcare teams, anchored by one acute hospital in the north and one acute hospital in the central. Hence, the region where the participants stayed in were also adjusted in the multiple logistic regression. All analyses were performed using Stata/SE 17.0. P < 0.05 was set as the level of significance.

The reporting of this study followed the STROBE checklist for cross-sectional studies (Appendix 1).

### Ethical considerations

The Population Health Index Survey was approved by the ethical committee of the institutional review board (Reference Number: 2015/00269). Written informed consent was obtained from each participant after being informed about the study purpose and confidentiality of the data collected.

## Results

### Characteristics of study participants

The mean age of the 1,528 participants was 63.3 years (SD = 13.5, range 21–98 years). Majority of the participants were Chinese (75.5%), living with others (81.0%), having personal income of ≤ SGD1,000 (53.0%), not smoking (88.2%) and having no alcohol abuse (87.9%). Seven hundred and sixty-six participants (50.1%) were young adults aged 21–64 years old (mean age ± SD: 52.6 ± 9.5) and 772 (49.9%) were older adults aged ≥ 65 years old (mean age ± SD: 74.1 ± 6.7). Participants’ characteristics stratified by group are presented in Table 1.

**Table 1:** Characteristics of study participants stratified by groups

Characteristics	Total N = 1528	Young adults (21–64 years) n = 766	Older adults (≥ 65 years) n = 762	p-value
**Age**, mean ± SD	63.3 ± 13.5	52.6 ± 9.5	74.1 ± 6.7	< 0.001
**Region**				< 0.001
Central	864 (56.5)	353 (46.1)	511 (67.1)	
Northern	664 (43.5)	413 (53.9)	251 (32.9)	
**Gender**, n (%)				0.004
Male	660 (43.2)	359 (46.9)	301 (39.5)	
Female	868 (56.8)	407 (53.1)	461 (60.5)	
**Ethnicity**, n (%)				< 0.001
Chinese	1153 (75.5)	525 (68.5)	628 (82.4)	
Malay	191 (12.5)	119 (15.5)	72 (9.5)	
Indian	151 (9.9)	97 (12.7)	54 (7.1)	
Others	33 (2.2)	25 (3.3)	8 (1.1)	
**Personal income level**, n (%)				< 0.001
>$4000	299 (19.6)	209 (27.3)	90 (11.8)	
$1001 to $4000	419 (27.4)	311 (40.6)	108 (14.2)	
≤$1000	810 (53.0)	246 (32.1)	564 (74.0)	
**Living alone**, n (%)				< 0.001
No	1237 (81.0)	668 (87.2)	569 (74.7)	
Yes	291 (19.0)	98 (12.8)	193 (25.3)	
**Employment status**, n (%)				< 0.001
Not employed	800 (52.4)	219 (28.6)	581 (76.3)	
Employed	728 (47.6)	547 (71.4)	181 (23.8)	
**Current smoking status**, n (%)				< 0.001
Not smoking	1347 (88.2)	639 (83.4)	708 (92.9)	
Smoking	181 (11.9)	127 (16.6)	54 (7.1)	
**Alcohol abuse (6 or more servings)**, n (%)				< 0.001
No	1343 (87.9)	641 (83.7)	702 (92.1)	
Yes	185 (12.1)	125 (16.3)	60 (7.9)	
**Presence of DHL**, n (%)				< 0.001
No	260 (17.0)	177 (23.1)	83 (10.9)	
Yes	1268 (83.0)	589 (76.9)	679 (89.1)	
**Number of medications**, n (%)				< 0.001
1 only	536 (35.1)	347 (45.3)	189 (24.8)	
2 or more	992 (64.9)	419 (54.7)	573 (75.2)	
**Presence of depressive symptoms**, n (%)				0.069
No	1400 (91.6)	692 (90.3)	708 (92.9)	
Yes	128 (8.4)	74 (9.7)	54 (7.1)	

Column percentages were reported. DHL: diabetes, hypertension, and dyslipidaemia

Compared to young adults, older adults had more females, Chinese, more individuals with low personal income (≤ SGD1,000), living alone, unemployed, with presence of DHL, and taking two or more medications. Compared to older adults, young adults had higher proportion of individuals who were currently smoking and having alcohol abuse (Table 1).

### Presence and reasons for medication non-adherence

The overall prevalence of medication non-adherence was 30.4% in all participants, and 31.0% and 29.5% among participants in the central and northern region (p = 0.527), respectively. When breaking down by group, it was 38.4% in young adults, significantly higher than 22.3% in older adults (*p* < 0.001). The prevalence of unintentional and intentional non-adherence was 34.5% and 14.4% respectively in young adults, which was also higher than the corresponding prevalence (unintentional: 20.1% and intentional: 5.9%, both *p* < 0.001) in older adults.

The prevalence of medication non-adherence in young and older adults by characteristics is presented in Table 2. Young adults staying in the central region (43.1%), having higher personal income, being employed (41.5%), taking two or more medications (42.0%), and presenting depressive symptoms (59.5%) had higher prevalence of non-adherence. In older adults, those being employed (27.6%), having any DHL (23.7%), and presenting depressive symptoms (48.2%) had higher prevalence of non-adherence.

**Table 2:** Prevalence of medication non-adherence by characteristics in each group

Characteristics	Young adults (21–64 years) n = 766			Older adults (≥ 65 years) n = 762		
	Adherent, n (%)	Non-adherent, n (%)	p-value	Adherent, n (%)	Non-adherent, n (%)	p-value
**Overall medication adherence**	472 (61.6)	294 (38.4)		592 (77.7)	170 (22.3)	
Unintentional	502 (65.5)	264 (34.5)		609 (79.9)	153 (20.1)	
Intentional	656 (85.6)	110 (14.4)		717 (94.1)	45 (5.9)	
**Region**			0.014			0.712
Central	201 (56.9)	152 (43.1)		395 (77.3)	116 (22.7)	
Northern	271 (65.6)	142 (34.4)		197 (78.5)	54 (21.5)	
**Gender**			0.438			0.273
Male	216 (60.2)	143 (39.8)		240 (79.7)	61 (20.3)	
Female	256 (62.9)	151 (37.1)		352 (76.4)	109 (23.6)	
**Ethnicity**			0.752			0.194
Chinese	330 (62.9)	195 (37.1)		493 (78.5)	135 (21.5)	
Malay	70 (58.8)	49 (41.2)		56 (77.8)	16 (22.2)	
Indian	58 (59.8)	39 (40.2)		39 (72.2)	15 (27.8)	
Others	14 (56.0)	11 (44.0)		4 (50.0)	4 (50.0)	
**Personal income level**			0.037			0.368
>$4000	114 (54.6)	95 (45.5)		68 (75.6)	22 (24.4)	
$1001 to $4000	204 (65.6)	107 (34.4)		79 (73.2)	29 (26.9)	
≤$1000	154 (62.6)	92 (37.4)		445 (78.9)	119 (21.1)	
**Living alone**			0.932			0.850
No	412 (61.7)	256 (38.3)		443 (77.9)	126 (22.1)	
Yes	60 (61.2)	38 (38.8)		149 (77.2)	44 (22.8)	
**Employment status**			0.005			0.049
Not employed	152 (69.4)	67 (30.6)		461 (79.4)	120 (20.7)	
Employed	320 (58.5)	227 (41.5)		131 (72.4)	50 (27.6)	
**Current smoking status**			0.178			0.747
Not smoking	387 (60.6)	252 (39.4)		551 (77.8)	157 (22.2)	
Smoking	85 (66.9)	42 (33.1)		41 (75.9)	13 (24.1)	
**Alcohol abuse** **(≥ 6 servings)**			0.226			0.274
No	401 (62.6)	240 (37.4)		542 (77.2)	160 (22.8)	
Yes	71 (56.8)	54 (43.2)		50 (83.3)	10 (16.7)	
**Presence of DHL**			0.080			0.008
No	119 (67.2)	58 (32.8)		74 (89.2)	9 (10.8)	
Yes	353 (59.9)	236 (40.1)		518 (76.3)	161 (23.7)	
**Number of medications**			0.023			0.323
1 only	229 (66.0)	118 (34.0)		151 (79.9)	38 (20.1)	
2 or more	243 (58.0)	176 (42.0)		441 (77.0)	132 (23.0)	
**Presence of depressive symptoms**			< 0.001			< 0.001
No	442 (63.9)	250 (36.1)		564 (79.7)	144 (20.3)	
Yes	30 (40.5)	44 (59.5)		28 (51.9)	26 (48.2)	

Row percentages were reported. DHL: diabetes, hypertension, and dyslipidaemia

The frequency and percentage for each reason for medication non-adherence in each group is shown in Table 3. The top seven reasons (> 10%) were similar between young and older adults although the order varied slightly. In both groups, the top reason was “Afraid of developing drug dependence or worry about long-term effects of medications”, which was reported by 74.8% of young adults and 73.5% of older adults, respectively. It was followed by “Problems with taking medication at specific time” (24.8%) and “Just forgot” (20.0%) in young adults, whereas by “Took a number of medications several times a day” (24.1%) and “Problems with taking medication at specific time” (21.8%) in older adults. Across the 17 reasons, significant difference was only observed in the reason for “Do not know how to read or understand medication labels” with older adults reporting higher rate (8.8%) than young adults (3.7%).

**Table 3:** The frequency and percentage of reasons for medication non-adherence in each group

Domain	Reason for medication non-adherence	Total N = 1528	Young adults (21–64 years) n = 766	Older adults (≥ 65 years) n = 762	p-value
Patient	I was afraid of developing drug dependence or I worry about long-term effects of my medications	345 (74.4)	220 (74.8)	125 (73.5)	0.825
Therapy	I had problems with taking medication at specific time (e.g., with meal, on an empty stomach, not at home)	110 (23.7)	73 (24.8)	37 (21.8)	0.498
Patient	I just forgot	103 (22.2)	69 (23.5)	34 (20.0)	0.418
Therapy	I took several medications several times a day	90 (19.4)	49 (16.7)	41 (24.1)	0.052
Therapy	I wanted to avoid side effects	85 (18.3)	60 (20.4)	25 (14.7)	0.136
Patient	I did not see any benefit in taking the medication	80 (17.2)	51 (17.4)	29 (17.1)	1.000
Socioeconomic	The medication was too expensive	62 (13.4)	41 (14.0)	21 (12.4)	0.673
Therapy	My doctor frequently changed my therapy	39 (8.4)	23 (7.8)	16 (9.4)	0.604
Condition	I felt sad, down, or blue	38 (8.2)	24 (8.2)	14 (8.2)	1.000
Healthcare system	I had run out of medication (e.g., did not get refills on time, medication was not available)	37 (8.0)	27 (9.2)	10 (5.9)	0.286
Patient	I did not understand why I need to take this medication or why it is important to stick to the instructions	30 (6.5)	15 (5.1)	15 (8.8)	0.122
Therapy	My medication regimen was too complex (e.g., odd dosing timings, irregular number of daily doses, cut tablets, use inhalers, injections)	28 (6.0)	16 (5.4)	12 (7.1)	0.545
Patient	I did not know how to read or do not understand what is written on the medication labels	26 (5.6)	11 (3.7)	15 (8.8)	0.034
Healthcare system	My doctor did not involve me in my treatment choices	18 (3.9)	9 (3.1)	9 (5.3)	0.318
Condition	It was hard for me to swallow the pills I had to take	13 (2.8)	5 (1.7)	8 (4.7)	0.079
Condition	I had physical difficulty in opening / administering medications	12 (2.6)	6 (2.0)	6 (3.5)	0.370
Socioeconomic	I did not want other people to see me taking medication	11 (2.4)	5 (1.7)	6 (3.5)	0.222

### Factors associated with medication non-adherence

The results of multiple logistic regression analysis (Table 4) showed that in young adults, staying in the northern region (OR = 0.63, 95%CI: 0.46, 0.86), having monthly income of SGD1,000–4,000 (OR = 0.52, 95%CI: 0.36, 0.76), and being currently smoking (OR = 0.60, 95%CI: 0.39, 0.94) were associated with lower odds of medication non-adherence. Being employed (OR = 2.96, 95%CI: 1.78, 4.93), taking ≥ 2 medications (OR = 1.42, 95%CI: 1.04, 1.96), and presence of depressive symptoms (OR = 2.99, 95%CI: 1.77, 5.04) were positively associated with higher odds of medication non-adherence. In older adults, presence of any DHL (OR = 2.55, 95%CI: 1.21, 5.38) and presence of depressive symptoms (OR = 4.27, 95%CI: 2.36, 7.74) were positively associated with higher odds of medication non-adherence. Hence, presence of depressive symptoms was a risk factor for non-adherence in both young and older adults.

**Table 4:** Factors associated with medication non-adherence in each group

	Young adults (21–64 years) n = 766			Older adults (≥ 65 years) n = 762		
	OR	95% CI	p-value	OR	95% CI	p-value
**Region (Ref: Central)**						
Northern	0.63	0.46, 0.87	0.004	0.87	0.59, 1.27	0.471
**Female (Ref: Male)**	0.84	0.60, 1.18	0.317	1.39	0.93, 2.07	0.108
**Ethnicity (Ref: non-Chinese)**						
Chinese	0.75	0.53, 1.05	0.095	0.77	0.49, 1.22	0.269
**Personal income level (Ref:>SGD4,000)**						
SGD1,001–4,000	0.53	0.36, 0.77	0.001	0.99	0.48, 2.02	0.974
<=SGD1,000	1.26	0.76, 2.10	0.376	0.84	0.47, 1.48	0.539
**Living alone**	0.89	0.55, 1.42	0.623	0.95	0.63, 1.43	0.819
**Employed**	2.92	1.76, 4.84	< 0.001	1.60	0.94, 2.74	0.084
**Currently smoking**	0.61	0.39, 0.95	0.030	1.12	0.55, 2.29	0.755
**Alcohol abuse**	1.25	0.81, 1.93	0.312	0.78	0.37, 1.63	0.511
**Presence of any DHL**	1.36	0.93, 1.99	0.111	2.63	1.25, 5.53	0.011
**Taking ≥ 2 medications**	1.42	1.04, 1.95	0.029	1.07	0.69, 1.64	0.774
**Presence of depressive symptoms**	3.00	1.78, 5.05	< 0.001	4.16	2.31, 7.51	< 0.001

95%CI: 95% confidence interval. DHL: diabetes, hypertension, and dyslipidaemia; OR: odds ratio; ref: reference

## Discussion

This cross-sectional study compared the prevalence and reasons for medication non-adherence in young and older adults and examined the association between potential risk factors and medication non-adherence in each group using data extracted from a representative population health survey among community-dwelling adults in the central and northern regions of Singapore. The results showed that the prevalence of overall medication non-adherence in young adults (38.4%) was higher than that in older adults (22.3%). The main reasons for medication non-adherence in young adults were mainly patient- and therapy-related factors, which were generally identical to those identified in older adults. Presence of depressive symptoms was consistently associated with non-adherence in both groups.

The prevalence of overall medication non-adherence observed in this study (30.4%) was relatively higher than that reported among type 2 diabetes adult patients (21–27%) in United States [34] and lower than the pooled prevalence (42.6%) among people living with multimorbidity [35]. The prevalence observed in older adults (22.3%) was much lower than that reported in another Singapore study (60.0%) conducted among a convenient sample of community-dwelling adults aged ≥ 60 years [1]. This probably could be explained by the different sampling methods used for participant recruitment. Similar to the findings of a Japan study [11], our results also showed that young adults had much higher prevalence of intentional and unintentional medication non-adherence than older adults, and prevalence of unintentional non-adherence was more than two times higher than intentional non-adherence in both young and older adults.

Being afraid of developing medication dependence was the predominant reason for non-adherence in both young and older adults. Together with another patient-related factor - unawareness of the benefit of medications, both reflect a person’s insufficiency in knowledge and negative beliefs on medication. Our findings align with other studies which found that patients’ poor knowledge and negative beliefs on medication are strong predictors of medication non-adherence [9, 36], which highlights the importance of addressing patients’ concerns on prescribed medications [15].

The high percentage of forgetting taking medications in both young and older adults, on the other hand, accounts for the high prevalence of unintentional non-adherence. Although these reasons are categorized as patient-related factors, it does not mean that patients should hold sole responsibility for non-adherence as it reflects the necessity of improving health literacy of the population and implementing good reminder systems and strategies, both of which should be addressed with the collaborative multi-level efforts [18]. It is not surprising that higher proportion of older adults reported non-adherence due to issues of reading medication labels than young adults. The proportion of young adults reporting it, although relatively low (3.7%), also suggests that difficulty in reading / understanding medication labels could happen in young adults as well. However, we could not pursue in depth whether the difficulty in reading / understanding medication labels was related to low education or low health literacy because there were no data on participants’ education background or health literacy due to a lack of appropriate items in the survey.

Taking medication at specific time was the most highly rated therapy-related factor. This reflects the barriers encountered by individuals. As some medications are time-sensitive for a variety of reasons, taking them at specific time is essential for ensuring effectiveness and minimizing negative side effects. Failing to take time-sensitive medications as prescribed, also a type of medication non-adherence, can lead to several unintended consequences which may cause further non-adherence. As an individual-level factor, it could potentially be improved by interventions from individual and system levels with the support of artificial intelligence technology [37]. Echoing prior studies [15, 38] which reported that taking multiple medications was associated with higher medication non-adherence, our study also showed that taking a number of medications several times a day was one of the main reasons for medication non-adherence in both age groups (4th and 2nd top rated causes in young and older adults, respectively). However, the multiple logistic regression results showed that taking two or more medications was associated with medication non-adherence in young adults only, suggesting that taking multiple medications may not be a strong predictor of medication non-adherence in older adults after adjusting for other potential factors, which resonates with the local study on adults aged 60 and above [1].

As one of the social and economic factors, the cost of medications is always a valid concern when it comes to long-term medication adherence [22]. In the study population, the item regarding cost of medications was rated as the 7th of the reasons for non-adherence in both young and older adults. Another social factor, which is related to privacy consideration, was the least rated factor in this study, suggesting that potential stigma and social discrimination on medication taking might not be a concern to local adults. Socio-economic factors examined in the regression were more likely to be associated with non-adherence in young adults: employed young adults reported higher risk of non-adherence while young adults with personal income of SGD1,001–4,000 reported lower risk of non-adherence compared to those with income of > SGD4,000. It seemed that the impact of medication cost on non-adherence could not be fully alleviated by high personal income level as it was not associated with non-adherence in older adults and no trending effects on non-adherence was observed in young adults, after adjusting for other potential factors.

Our study found having depressive symptoms was consistently associated with higher risk of medication non-adherence in both young and older adults, which provides additional evidence from Asian perspective that depression has a negative impact on medication adherence [39]. Aligned with other studies [36, 40, 41], being diagnosed with any DHL corresponded to a higher risk of non-adherence in both age groups although it was only significant in older adults in this study. This probably could be explained by the difference in prevalence, severity and impact of DHL as well as disease perceptions in the two age groups.

In contrast to the studies reporting that current smoking was associated with medication non-adherence in young and older adults [1, 11], our study observed different impact of current smoking on non-adherence in the two groups: while there was no significant association observed in older adults, current smoking was surprisingly associated with lower risk of non-adherence in young adults. The reasons for the independent association between current smoking and lower medication non-adherence in this group are unclear. One possible explanation is the difference in classification criteria for current smoking compared to previous studies. Another potential explanation is that unmeasured variables like health literacy and health activation could mediate the association between smoking and non-adherence [42]. Further research is needed to explore these explanations and gain a comprehensive understanding of this relationship.

This study has several strengths. First, the participants were sampled from a representative population health study, hence, they were more likely to be representative of the community-dwelling adult population with chronic conditions. Second, this study adopted WHO’s system model for adherence and examined the reasons for non-adherence based on local context. Therefore, it is possible that the findings of the study are practical to guide the intervention and strategy development for improving medication adherence. There are also a few potential limitations for the study. Firstly, the data on medication non-adherence used in the study were collected by retrospective recall using survey questionnaire during face-to-face interviews, the accuracy and reliability of the data collected was subject to recall error. Secondly, some participants might be less likely to report non-adherence or under report the frequency of medication non-adherence due to social desirability bias. Hence, the prevalence calculated in the study might be lower than the actual prevalence. However, due to the study design, we could not validate the accuracy and reliability as we did not monitor drug intake through lab tests, or collect insurance claims and pharmacy data (e.g., drug refill record or pill counts). Thirdly, the medication non-adherence instrument used in this study was revised from existing measures with some culture adaption and revision. Hence, the prevalence and reasons for medication non-adherence derived from the study might not be directly comparable to other studies.

Our findings could potentially guide healthcare professionals including community pharmacists and nurses in designing tailored strategies or interventions to improve medication adherence in different age groups, such as implementing patient education and support programs to improve their awareness of the importance of medication adherence for chronic disease management, addressing medication concerns and management of side effects, pasting medication labels in the preferred language (in addition to English language) for non-English speaking older adults [43], and developing automated text messaging programs to send medication reminders with the assistance of current technologies. The consistent association between presence of depressive symptoms and medication non-adherence in both young and older adults suggest that poor mental health or mental state might be a risk factor of non-adherence. Hence, additional strategies for medication adherence should be developed for patients with depressive symptoms or mental health concerns.

## Conclusions

The reported medication non-adherence rate in young adults was 16.1% higher than that in older adults in the community setting. The main reasons for non-adherence reported by young and older adults were generally comparable. Presence of depressive symptoms was a risk factor of medication non-adherence in both groups. Our findings could potentially guide healthcare professionals in designing tailored strategies or interventions to improve medication adherence in community-dwelling patients of different age groups.

### Electronic supplementary material

Below is the link to the electronic supplementary material.

**Supplementary Table 1**. The list of 17 potential causes and their domains.

## Data Availability

According to the Data Protection Act Commission Singapore-Advisory Guidelines for the Healthcare Sector, all the individual data collected for the Population Health Index study are protected under the Act. As such, the datasets analysed during the current study are not publicly available. However, minimal dataset underlying the findings in the manuscript is available from the corresponding author on reasonable request.
