# Supplementary material for: Understanding reasons and determinants of medication non-adherence in community-dwelling adults: a cross-sectional study comparing young and older age groups
Source: BMC Health Serv Res. 2023 Aug 24;23:905. doi: 10.1186/s12913-023-09904-8 (PMC10464472; doi:10.1186/s12913-023-09904-8)
Supplement: Supplementary file 1 — Supplementary Table 1. The list of 17 potential causes and their domains. [file 12913_2023_9904_MOESM1_ESM.docx]

Supplementary Table 1. The list of 17 potential causes and their domains

| **SN** | **Domain** | **Cause of medication non-adherence**  *Each cause has four options: “Never”, “Rarely (1-2 times yearly)”, “Sometimes (3-5 times yearly)”, and “Often (>5 times yearly)”.* |
| --- | --- | --- |
| 1 | Patient-related | I did not see any benefit in taking the medication |
| 2 | Patient-related | I did not know how to read or do not understand what is written on the medication labels |
| 3 | Patient-related | I did not understand why I need to take this medication or why it is important to stick to the instructions |
| 4 | Patient-related | I was afraid of developing drug dependence or I worry about long-term effects of my medications |
| 5 | Patient-related | I just forgot |
| 6 | Therapy-related | I had problems with taking medication at specific time (e.g., with meal, on an empty stomach, not at home) |
| 7 | Therapy-related | I took several medications several times a day |
| 8 | Therapy-related | My medication regimen was too complex (e.g., odd dosing timings, irregular number of daily doses, cut tablets, use inhalers, injections) |
| 9 | Therapy-related | I wanted to avoid side effects |
| 10 | Therapy-related | My doctor frequently changed my therapy |
| 11 | Condition-related | I felt sad, down, or blue |
| 12 | Condition-related | I had physical difficulty in opening / administering medications |
| 13 | Condition-related | It was hard for me to swallow the pills I had to take |
| 14 | Healthcare system-related | My doctor did not involve me in my treatment choices |
| 15 | Healthcare system-related | I had run out of medication (e.g., did not get refills on time, medication was not available) |
| 16 | Social/economic | I did not want other people to see me taking medication |
| 17 | Social/economic | The medication was too expensive |
